# Amorphous cellulose nanofiber supercapacitors with voltage-charging performance

**DOI:** 10.1038/s41598-022-09649-0

**Published:** 2022-04-04

**Authors:** Mikio Fukuhara, Tomonori Yokotsuka, Toshiyuki Hashida, Tamon Miwa, Nobuhisa Fujima, Masahiro Morita, Takeshi Nakatani, Fuminari Nonomura

**Affiliations:** 1 New Industry Creation Hatchery Center, Tohoku University, Sendai, 980-8579 Japan; 2 Graduate School of Engineering, Fracture and Reliability Research Institute, Tohoku University, Sendai, 980-8579 Japan; 3 Faculty of Engineering, Shizuoka University, Hamamatsu, 432-8561 Japan; 4 Fuji Innovative Materials Research Laboratory, Nippon Paper Industries, Co. Ltd., Fuji, 417-8520 Japan

**Keywords:** Biophysics, Biotechnology, Materials science

## Abstract

The electric charge storage properties of amorphous cellulose nanofiber (ACF) supercapacitors with different metal carboxylate radicals (COOM; M: Na(I), Ca(II), Al(III)) was investigated in terms of charging/discharging behaviours, alternating current impedance analysis, and plane-wave-based first-principles density functional calculations. Na-ACF exhibited a higher storage effect than Ca- and Al-ACFs. The charge storage mechanism for an Na-ACF supercapacitor was proposed using an electric double layer model in a C_12_H_17_O_11_Na electrolyte with an electrical resistivity of 6.8 × 10^3^ Ω cm, based on the migration of protonic soliton. The supercapacitor, which demonstrated fast charging upon voltage application, could illuminate a white LED for 7 s after charging with 10 mA at 18.5 V.

## Introduction

Following the discovery of amorphous titanium-oxide (golden, a-TiO_2−x_, ATO)^1^, perfluorinated polymer (APP)^2^, and aluminum-oxide (blackish, a–Al_2-y_O_3−z_, AAO) supercapacitors^3,4^, we identified an amorphous cellulose nanofiber (ACF) supercapacitor that can store a large amount of electricity (221 mJ m^−2^, 13.1 Wkg^−1^)^5^ (Supplementary Information [hereafter, referred to as (SI)] Fig. S1). The electrical storage can be attributed entirely to the enhanced electroadsorption, i.e., the work function of − 22.5 eV at 17.9 nm, because of a quantum-size effect induced by a convexity of 17.9 nm, an offset effect caused by positive polar C_6_=O_6_ radicles, and an electrostatic effect owing to localized electrons near the Na ions. The supercapacitor could capture both positive and negative charges from the atmosphere and in a vacuum and could illuminate a red light-emitting diode (LED) for 1 s after charging it with a current of 2 mA at 10 V. The ACF, composed of natural products that are environmentally friendly, is a pioneer in paper electronics with potential applications in light electricity. Furthermore, the ACF supercapacitor is completely different from conventional “wet” cells, such as electric double-layer capacitors (EDLCs) and lithium-ion batteries (LIBs), wherein the charge is controlled by the diffusivity of ions^6,7^. Here, we report the electronic role of the sodium carboxylate (COONa) radical and the mechanism for the electric storage of ACF supercapacitors, with the aim of obtaining greater electric charge storage by fast charging under voltage application.

## Results and discussion

### Electric storage system of ACF

**Figure 1:**
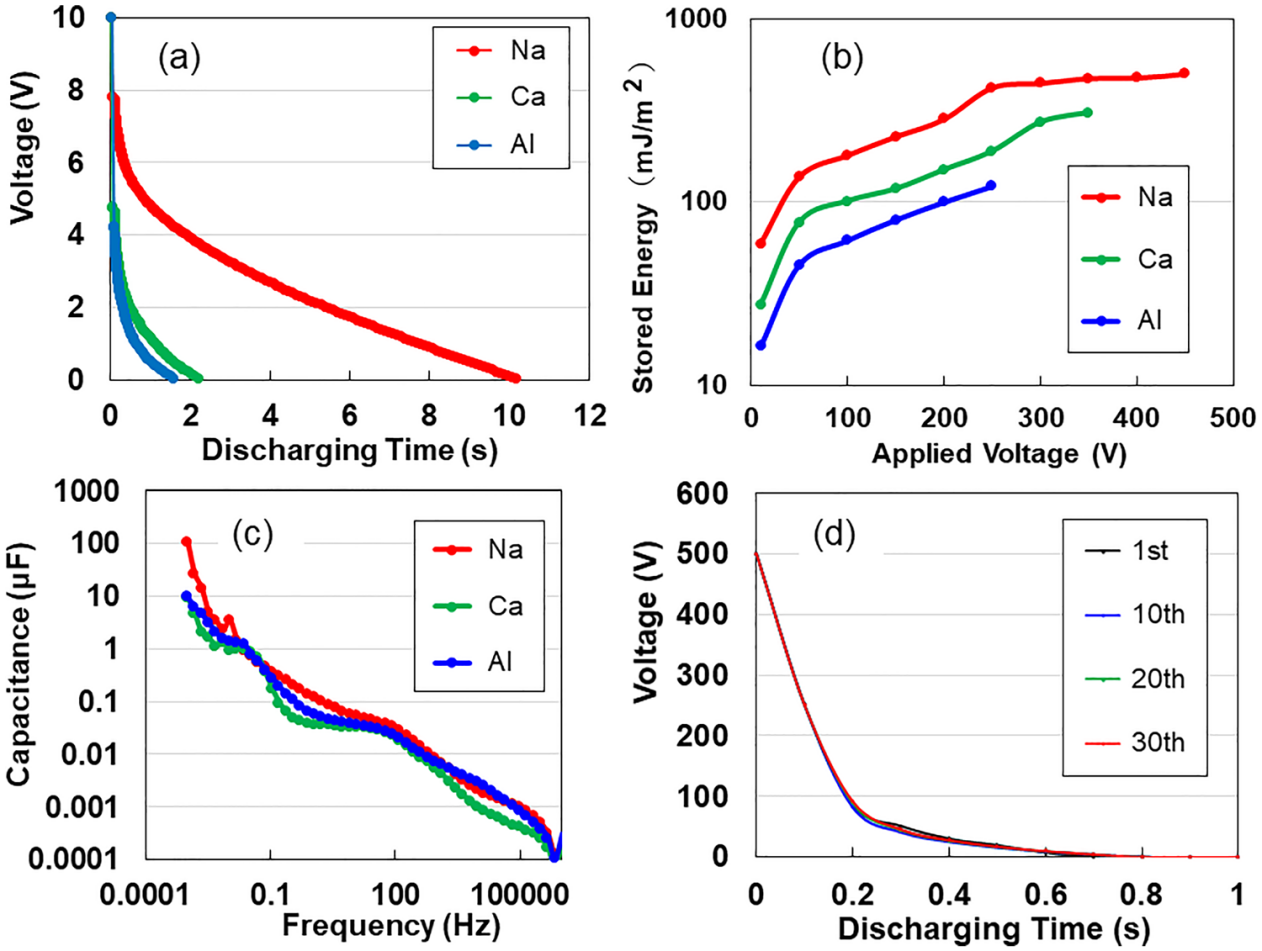
Electric behaviours for Na-, Ca-, and Al-ACF devices. (**a**) The discharging behaviours for a constant current of 1 μA after 2 mA-10 V charging for 50 s. (**b**) Applied voltage dependency of stored energy. (**c**) Series capacitance as a function of frequency. (**d**) Discharging time for 2 mA-5 s charging/1 μA-discharging up to 30 times.

Shimizu et al.^8^ reported the counterion exchange from once-dried TOCN-COONa to TOCNs-COOM radicals (M: groups I, II, and III metal ions). We compared the DC discharging behaviours of Ca(II)- and Al(III)-ACFs with that of Na(I)- with a surface area of 1.8 cm^2^ and thickness of 3 μm under a constant current of 1 μA. Figure 1a shows the discharging curves of ACF devices after 2 mA—10 V charging for 50 s. The Na-ACF specimens exhibited discharging behaviours with electric storage that was four and six times greater than those of the Ca- and Al-ACFs, respectively. All curves show an ohmic *IR* drop of up to 1.5, 5.4, and 6.1 V for Na-, Ca-, and Al-ACFs, respectively, through gradual reduction. The *IR*-drop was attributed to the internal charging of unsaturated cells as well as the EDLC^9^. Contrary to the conventional long-time charging of EDLCs and LIBs under a constant current, the ACF supercapacitor demonstrated short-time charging upon voltage application. As per Fig. 1b, the Na-ACF exhibits a superior storage effect (~ 503.6 mJ m^−2^) up to 450 V, although the high-voltage application of the Ca- and Al-ACFs is limited by its feature. A previous study^5^ for Na-ACF reported the electricity storage capacity of 221 mJ m^−2^. The discharging behaviours of other M-ACFs are presented at SI Fig. S2. Figure 1c shows the frequency-dependent series *Cs* capacitance. The *Cs* of Na-ACF exceeded those of Ca- and Al-ACFs at all frequencies. Therefore, the Na-ACF was selected as an ideal supercapacitor. We repeated the test under 2 mA—5 s charging/1 μA-discharging up to 30 times at 500 V, using a specimen with 1.2 cm (wide) × 1.5 cm (length) × 20 μm (thickness). Figure 1d indicates short-time charging without *IR* drop at a voltage that is 100 times greater than those of EDLCs and LIBs.

### Optimized structure of ACF and their electronic role

**Figure 2:**
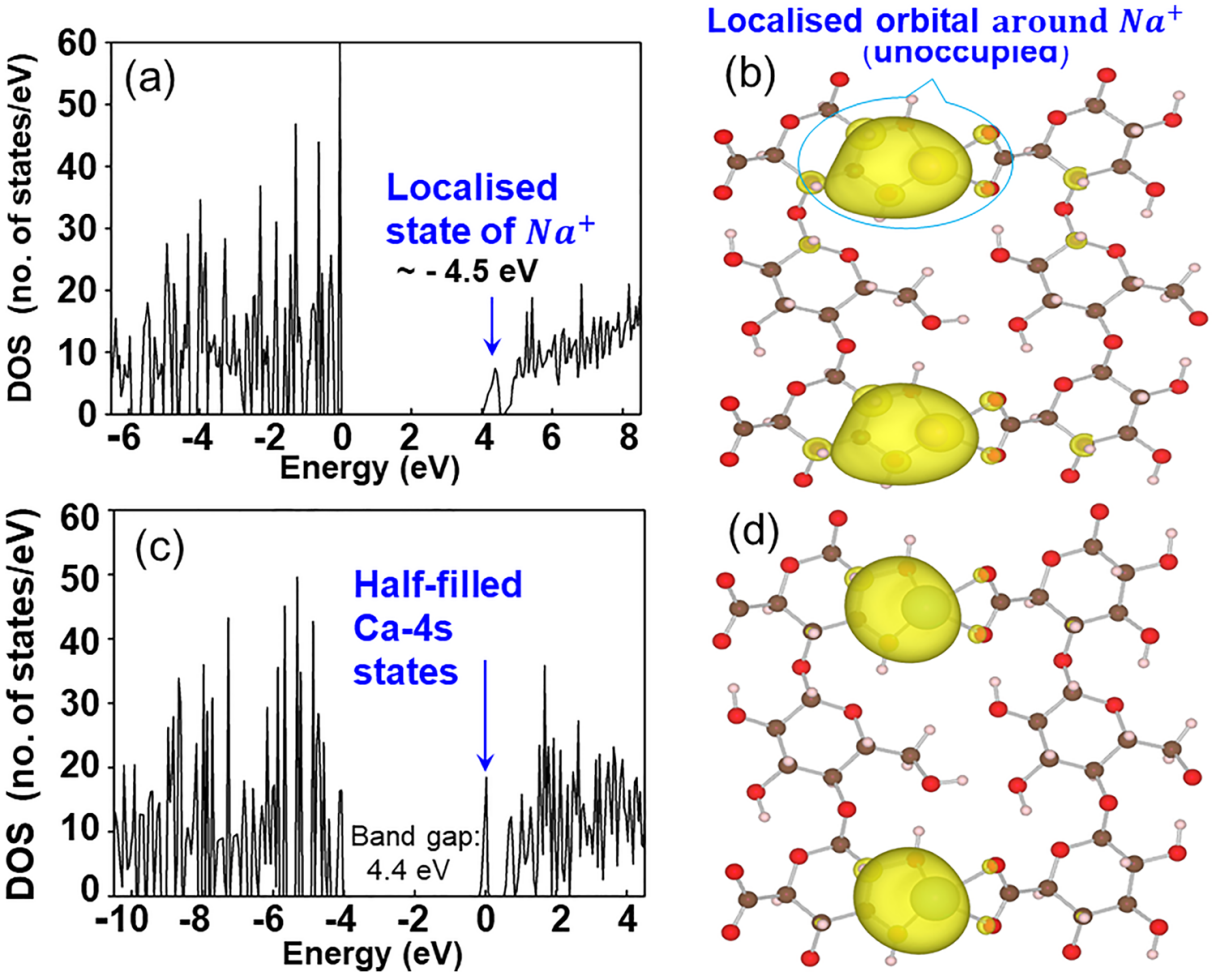
(**a**) DOS in COONa-cellulose sheet. (**b**) Density distribution of localized state in COONa-cellulose sheet. (**c**) DOS in COOCa-cellulose sheet. (**d**) Density distribution of LUMO in COOCa-cellulose sheet.

To determine the reasons underlying the superior electric adsorption of Na-ACF, we optimized the local structures around COONa, COOCa, and COOAl radicals of TEMPO-oxidized cellulose sheets, TOCN-COONa, -COOCa, and COOAl, using first-principles calculations. We then simulated the density of states (DOS) for C_12_H_17_O_11_Na, C_12_H_17_O_11_Ca, and C_12_H_17_O_11_Al units. The DOSs in the C_12_H_17_O_11_Na, C_12_H_17_O_11_Ca, and C_12_H_17_O_11_Al celluloses are depicted in Figs. 2a,c and Suppl Fig. S4a, respectively. Similar to the DOS of C_12_H_17_O_11_Na with COONa radical (Fig. 2a), the localized state of the 4 s electron state of the Ca cation in COOCa radicals appeared at 0.5 eV below the conduction band in the band gap for C_12_H_17_O_11_Ca (Fig. 2c). This localized state of the 4 s electron corresponded to the empty orbital of the 3 s electron of the Na cation, as shown in Fig. 2b. However, the localized electronic state in C_12_H_17_O_11_Ca in the density distribution of LUMO (Fig. 2d) was half-occupied, with one electron and one vacancy, because of the divalent 4 s electron in Ca. The result of C_12_H_17_O_11_Al cellulose is explained in Fig. S4. Per our previous study^5^, we inferred that the presence of a localized electron in the vicinity of the COONa radical induces positive charges (electrostatic effect) within the insulating ACF surface, leading to the high adsorption of many electrons from the atmosphere and in the vacuum. Therefore, we confirm that the localized electrons in the COONa radical have a greater effect on electric storage than those in COOCa and COOAl radicals. Therefore, we considered an electric storage mechanism using Na-ACF.

### Complex evaluation of electric storage and *I–V* characteristics

**Figure 3:**
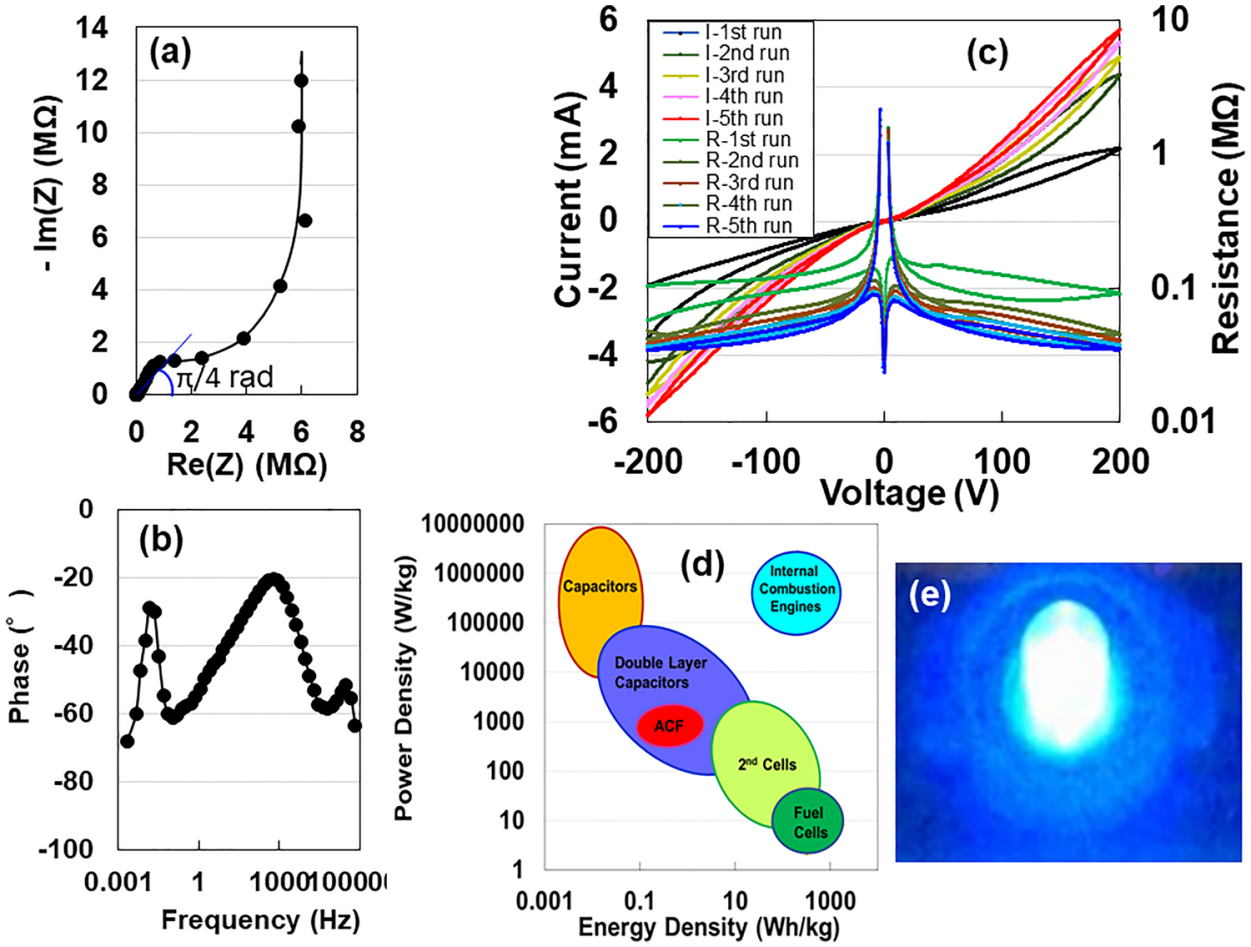
(**a**) Nyquist plot as a function of frequency for the ACF device. (**b**) Frequency dependence of phase angle. (**c**) *I–V* and *R–V* characteristics between − 200 and + 200 V up for five charge–discharge cycles. (**d**) Comparison of power density and energy density for ACF (this study), capacitors, EDLC, 2nd and fuel cells, and internal combustion energies (based on Whittingham’s study^14^). (**e**) An LED powered by the ACF device.

To non-destructively analyze the electrostatic contribution of the specimen after charging and discharging at 400 V, we measured the AC impedance from 1 Hz to 1 MHz at a constant voltage of 10 mV. We present a complex-plane (Nyquist) plot of the impedance data in Fig. 3a. The frequency dependent impedance is presented by the combined pattern of a line with a slope of π/4 rad (Warburg region) and a straight vertical line^2–5^. This indicates that the electrode is an ACF film with a porous surface characterized by a series RC circuit, as well as a graphene EDLC^10^. As the straight line starts at 6.0 MΩ in Fig. 3a, the resistivity of the electrode can be calculated as ρ = 6.8 × 10^3^ Ω cm (= 6. 0 × 10^6^ Ω × 1.2 cm (wide) × 0.0016 cm (thickness)/1.7 cm (length)). This value is within the range of resistivities of prospective sulfide solid electrolytes for solid-state lithium batteries^11^. The reduction in phase angle to − 90°with a decreasing frequency provides aditional evidence of direct current (DC) charging (Fig. 3b). Figure 3c represents the double *I–V* and *R–V* characteristics repeated five times using a DC method between − 200 and + 200 V in air at 293 K. The *I–V* curves exhibit nonlinear electronic transportation behaviour, which is similar to the Coulomb blockade behaviour observed in a metal–semiconductor junction^12^. The *I–V* curves are almost symmetric with respect to zero bias. By repeating up to five times, the current increases and the resistance decreases as the voltage increases. This implies behaviour that is similar to that of a pair of conductors, each comprising an electron and a positive hole (SI; Fig. S6). Indeed, positron annihilation lifetime spectroscopy of Na-ACF revealed an average pore size of ~ 0.47 nm in the region spanning from the film surface to depths of up to ~ 2 μm^13^. Then, we calculated power density and energy density for the specimens stored at 2 mA and ~ 450 V. The Ragone plot for the ACF supercapacitor is presented in Fig. 3d, along with those for conventional capacitors, EDLCs, and the second and fuel cells^14^, indicating a similar capability to that of EDLC. We illuminated a white LED to prove the electric energy storage properties of ACF. The device with a surface area of 100 cm^2^ and depth of 10 μm, lit the LED for 7 s (Fig. 3e) after it was charged with 10 mA at 18.5 V charging for 10 s (SI; Fig. S8).

### Mechanism for the electric storage of ACF

**Figure 4:**
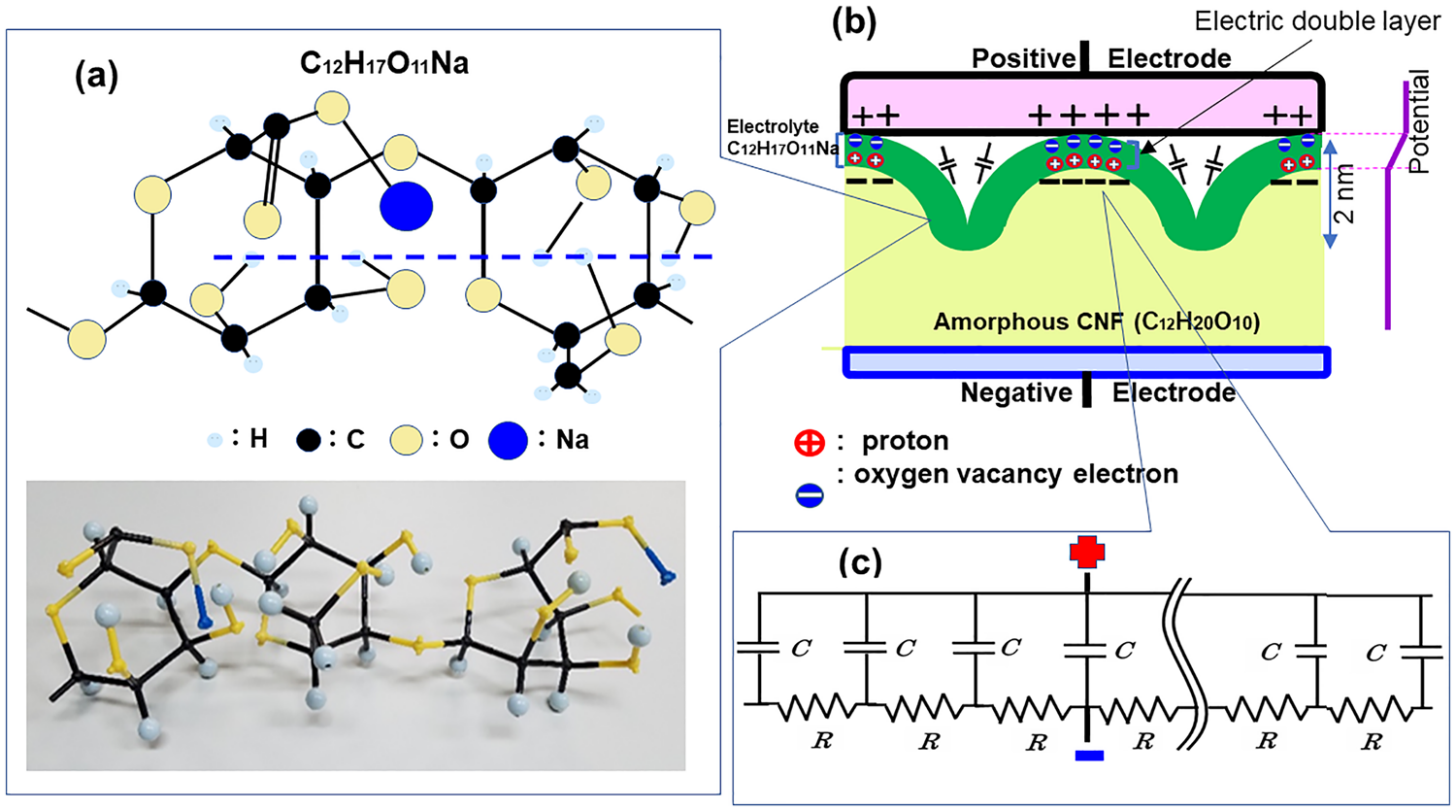
(**a**) Kink structure of C_12_H_17_O_11_Na molecules with COONa radical in TEMPO-oxidized cellulose nanofibril (TOCN) films with carbon in black, oxygen in orange, hydrogen in gray, and sodium in blue. (**b**) Schematic of the microscopic electric energy storage, based on electric double layer formed in C_12_H_17_O_11_Na electrolyte. (**c**) The electric distributed circuit of the ACF surface.

When the TEMPO-mediated oxidation was applied to native celluloses comprising amorphous microfibrils of cellulose, the primary hydroxy groups at C_6_ exposed on the microfibril surfaces were entirely oxidized to COONa^15^. The carboxylated cellulose microfibril was covered with glucose/glucuronic acid alternating with co-polysaccharide molecules. The surface structure of ACF with carbon, oxygen, hydrogen, and sodium atoms is schematically presented in Fig. 4a. All hydroxy groups were arranged along the equatorial direction of the dotted line to its chair conformation. The kink molecular structure of the oxidized surface was characterized by electrolyte C_12_H_17_O_11_Na with an electric resistivity of 6.8 × 10^3^ Ω cm. The dynamics of protons in a one-dimensional hydrogen-bonded chain are currently drawing considerable attention in the field of biophysics^16–18^. Sugawara et al.^19^ observed a characteristic temperature- and frequency-dependent dielectric response derived from the solitonic migration of ionic defects generated by local proton transfer between molecules. Protonic solitons, which move as an isolated wave from the billiard movement of protons in ionic defective species, provide relatively low energies for the movement of protons and charges in hydrogen bond chains^20^. Hence, by analogy, we infer that the transferred protons align on the concave surface of the amorphous microfibril C_12_H_20_O_10_ surface. Figure 4b shows a morphological schematic that suggests a possible mechanism for large electrical charges by the formation of a pair comprising an electron and a proton. The protonic soliton migrates along the one-dimensional hydrogen-bonded kink chains. The localized electrons in the vicinity of the COONa radical induced by the positive electrode group together on the concave portion of the ACF. Therefore, the electrons and protons form a pair to maintain electric charges. In Fig. 4b, a line of pairs in the green cover region of the ACF is called an electric double layer (EDL) in the C_12_H_17_O_11_Na electrolyte. The amount of electricity stored in the EDL can abruptly discharge through native cellulose (C_12_H_20_O_10_) via a negative electrode, based on the current controlled by a variable resistor or an invertor of the outer circuit^21^. Therefore, the electric distributed-constant equipment circuit is organized on the amorphous Na-ACF surface (Fig. 4c). The uneven surface serves as an EDC circuit with an electrolyte layer containing nanometre-sized capacitors throughout the bulk.

## Conclusions

The AC capacitance and DC charging/discharging behaviours of Na-, Ca-, and Al-ACF were measured to determine a clear electronic contribution to the electric storage of ACF supercapacitors. The Na-ACF exhibited superior electric storage compared with Ca- and Al-ACF, based on the appearance of the localized electrons near the Na ions. The EDL model for electrolyte C_12_H_17_O_11_Na provides useful information for the material design of promising biomaterials. The Ragone plot of the ACF supercapacitor indicates a similar capability to that of the EDLC.

## Methods

The 2,2,6,6-tetramethylpiper-idine-1-oxyl (TEMPO-) oxidized carbon nanofibers (CNFs) (sodium carboxylate (COONa) content: 1.48 mmol g^−1^) with a diameters of 3-nm were prepared by Nippon Paper Industries. The alternating current (AC) capacitance and direct current (DC) charging/discharging behaviours were analysed using galvanostatic charge/discharge through a potentiostat/galvanostat (SP-150, BioLogic Science Instruments) with a DC of 10 V, 1 μA for ~ 60 s and charging current of 2 mA for 50 s at 293 K. The energy stored under the application of voltage ranging from 10 to 450 V was determined using a DC voltage current source/monitor (G247G, ADCMT). The optimized local atomic configurations of C_12_H_20_O_10_, C_12_H_17_O_11_Na, C_12_H_17_O_11_Ca, and C_12_H_17_O_11_Al units were determined using plane-wave-based first-principles density functional calculations (VASP 5.3)^22^.

## Supplementary Information

Supplementary Information.
