# Supplementary material for: Amorphous cellulose nanofiber supercapacitors with voltage-charging performance
Source: Sci Rep. 2022 Apr 4;12:5619. doi: 10.1038/s41598-022-09649-0 (PMC8980078; doi:10.1038/s41598-022-09649-0)
Supplement: Supplementary file 1 — Supplementary Information. [file 41598_2022_9649_MOESM1_ESM.docx]

**SUPPLEMENTARY INFORMATION**

**Amorphous cellulose nanofiber supercapacitors with voltage-charging performance**

Mikio Fukuhara,^1*^ Tomonori Yokotsuka,^1^ Toshiyuki Hashida,^2^ Tamon Miwa,^3^ Nobuhisa Fujima,^3^ Masahiro Morita,^4^ Takeshi Nakatani, ^4^ and Fuminari Nonomura ^4^

^1^ New Industry Creation Hatchery Center, Tohoku University, Sendai 980-8579, Japan,

^2^ Fracture and Reliability Research Institute, Graduate School of Engineering, Tohoku University, Sendai 980-8579, Japan,

^3^ Faculty of Engineering, Shizuoka University, Hamamatsu 432-8561, Japan,

^4^ Fuji Inovative Materials Research Laboratory, Nippon Paper Industries, Co. Ltd., Fuji 417-8520, Japan,

**S1. Methods**

Once-dried bleached hardwood kraft pulp fibers were used as the cellulose source for 2, 2, 6, 6-tetramethylpiperidine-1-oxyl radical (TEMPO)-mediated oxidation at 293 K under aqueous conditions. TEMPO-mediated oxidation and subsequent mechanical distribution were performed according to a previous reported method^8,21^. The resulting TEMPO-oxidized cellulose nanofiber (TOCN), with the H ions of its carboxyl groups replaced by sodium, lithium or potassium ions (-COONa, -COOLi or -COOK), had an average diameter of 3 nm and carboxyl content of 1.5 mmol/g. The 0.5% (w/v) TOCN/water dispersion was converted to a film with approximately 5 μm thickness. A 5-μm-thick ACF specimen was fabricated on an Al substrate by electrophoresis method at 10 V and ～0.01 A for 120 s. The TOCN films obtained were dried in a ventilated oven at 323 K overnight. After TEMPO-oxidized CNFs (COONa content: 1.48 mmol/g, TC-01A) with 3 -nm diameters were prepared, the dried TEMPO-oxidized cellulose nanofibril (TOCN)- COONa films were soaked in an aqueous metal chloride solution to change the counterion to TOCN-COOM (M: group I, II and III metal)^8^. The specific surface area was measured by N adsorption, using of BET absorption (Gemini VII 2390, Shimadzu) at 378 K. Sample structure was examined by X-ray diffraction (XRD-7000X, Shimadzu). The surface morphologies were analyzed by atomic force microscope (AFM: NC-AFM, JSPM-5200, JEOL).

Because the discharging curves are not generally straight line, the amount of energy stored (*E*) can be calculated from the integral value of the *V* (voltage)-*t* (time) curve under a constant current (*I*): *E* = *I*×$\sum_{t=0}^{t=n} VI$(Joule), where *n* is a measurement point when *V* = 0.

**S2. Surface analyses of Na-ACF**

We investigated the structural morphologies and surface characteristics of the Na-ACF specimen. Figure S1a and b show an atomic force microscopy (AFM) image and three-dimensional AFM one of the specimen surface, respectively. The fibrous appearance of the outer-surface resembles the uneven surface of ATO^1^, APP^2^, and AAO^3,4^ (SI Fig. S6 in Ref. 5). The wide-field X-ray diffraction pattern (Fig. S1c) shows that the specimen consists of an amorphous cellulose phase, characterized by two broad peaks at approximately 16 and 22°. Although few nanocrystals were recognized from the continuous Debye rings in the selected-area electron diffraction pattern (insert Fig.3a in Ref. 5), the nanocrystal is distinguished from a perfect crystal. In general, amorphous materials are composed of subnanometre-sized clusters.^22, 23^ Because crystallisation from amorphous phases requires large activation energies such as heat, pressure, electron emission, *etc*., it is hard to crystalise for cellulose molecules at room temperature. On the other hand, an electron diffraction analysis of amorphous celluloses is not necessarily suitable, because the crystallisation of amorphous materials can be attributed to electron-beam irradiation^24^.

Fig. S1. (a) AFM image of the Na-ACF surface. (b) Three-dimensional AFM image. (c) XRD analysis of Na-ACF specimen.

**S3. Electric storage effect for Na-, Ca-, Al-, Li-, Mg- and Cu-ACFs**

We investigated an effect of applied voltage for electric storage, using Na-, Ca-, Al-, Li-, Mg-, K- and Cu-ACFs. These results are presented at Fig. S2. All specimens other than Na-ACF leaked above 50 V for Cu-ACF, 250 V for Al-ACF, 300 V for Li- and Mg-ACFs, 350 V for Ca-ACF and 400 V for K-ACF by dielectric breakdown. Especially, the Cu-ACF is not suitable as an electrolyte, because it is evaluated as good ion conductors for solid-state batteries^25^.

However, the Na-ACF specimen could be repeated the test under 2 mA-rapid charging/1 μA-discharging up to 30 times at 500 V (see Fig. 1d).

s

Fig. S2 Applied voltage dependency of stored energy on Na-, Ca-, Al-, Li-, Mg-, K-, and Cu-ACFs, where their depths are 0.4, 1.8, 1.8, 1.8, 1.0, 3.0 and 1.8 μm, respectively.

**S4. Charging behaviours for Na-, Ca-, Al-ACFs**

Figure S3 shows the current curves of the ACF device for 2 mA-10 V charging for 50 s. After the voltage of the sound specimen reached to a constant value in a moment, the current abruptly decreased to the lowest value and then continued as the charging time increased, as shown in the Na- and Al-ACF specimens. The abrupt decrease of currents in charging run demonstrates rapid charging by voltage application. On the other hand, the Ca-ACF specimen gradually decreased as the charging time increased, suggesting a shortage of charging time.

**S5. Electronic role for Na-ACF.**

We optimized the local structures around COOAl (III) radicals in Al-AFC (C_12_H_17_O_11_Al) unit and then simulated the density of states (DOS) for C_12_H_17_O_11_Al. The DOS, the local structures and localized state (charge density) are depicted in Figs. S4a, b, respectively.

Fig. S3 The current curves of Na-, Ca- and Al-ACF for 2mA-10 V charging for 50 s.

Fig. S4 (a) Density of state (DOS) in C_12_H_17_O_11_Al unit. (b) Local structures and isolated electronic state (yellow) locally occur in vicinity of Al ion.

The DOS in Fig. S4a shows an isolated state in the lower side of the bandgap. This localized state is originated from the Al-3s electronic orbital, and the corresponding localized charge distribution appears around the Al cations as shown in Fig. S4b, similarly to those for Na-ACF and Ca-ACF in Figs. 2b and 2d. However, this localized 3s state is fully occupied by two electrons in contrast to the empty Na-3s state and to the half-filled Ca-4s state. Therefore, the Al-3s state hardly contributes the electronic storage. Moreover, the local structure in Fig. S4b shows an asymmetric (distorted) Al-carboxylate (COOAl) structure while the typical symmetric metal-carboxylate structures appear in COONa and COOCa in Figs. 2b and 2d. As a result, an unstable COO(-Al) state emerges below the bottom of the conduction band. This empty state possibly plays a minor role similar to the Na-3s state.

**S6. Complex evaluation of electric storage and *I ̶ V* characteristics.**

To analyse non-destructively the electrostatic contribution of the specimen, we measured the AC impedance from 1 mHz to 1 MHz. The imaginary and real impedances rapidly increase up to 10 and 6 MΩ in the lower-frequency region of the Bode diagram, respectively (Fig. S5). This is another evidence of DC charging.

From Fig. 3e, we obtained run’ number dependence on current and resistance at ± 200 V. Figure S6 shows that the *I–V* curves are almost symmetric with respect to zero bias and reveals the almost same resistance for positive and negative polarity independent of run’s number. Because the film surface (~ 2 nm in depth) of the Na-ACF is oxide phase with average pore size of ~0.47 nm^13^ and electric resistivity of 6.8 × 10^3^ Ωcm, it is regarded as an electrolyte phase with negative electrons and positive vacancies (holes) or protons. Here, we consider an electrical rectification effect for the electrolyte by switching of voltage. In a metal-semiconductor junction, when positive voltage applies to the metal electrode, the holes move across the junction from the semiconductor to the metal, the electrons migrate across the junction in the opposite direction into the region where there are large numbers of positive holes. Conversely, when positive voltage applies to the semiconductor, the semiconductor material has lost electrons and holes. This behaviour is well known as the rectification effect of the single-channel conductance. However, the rectification effect suppresses when the material consists of equivalent numbers of electrons and holes. Hence, we infer by analogy that the mechanism for electric storage of the Na-ACF device is determined by electric transfer of pairs of negative electron and positive hole (vacancy) or proton in surface region of Na-ACF sheet.


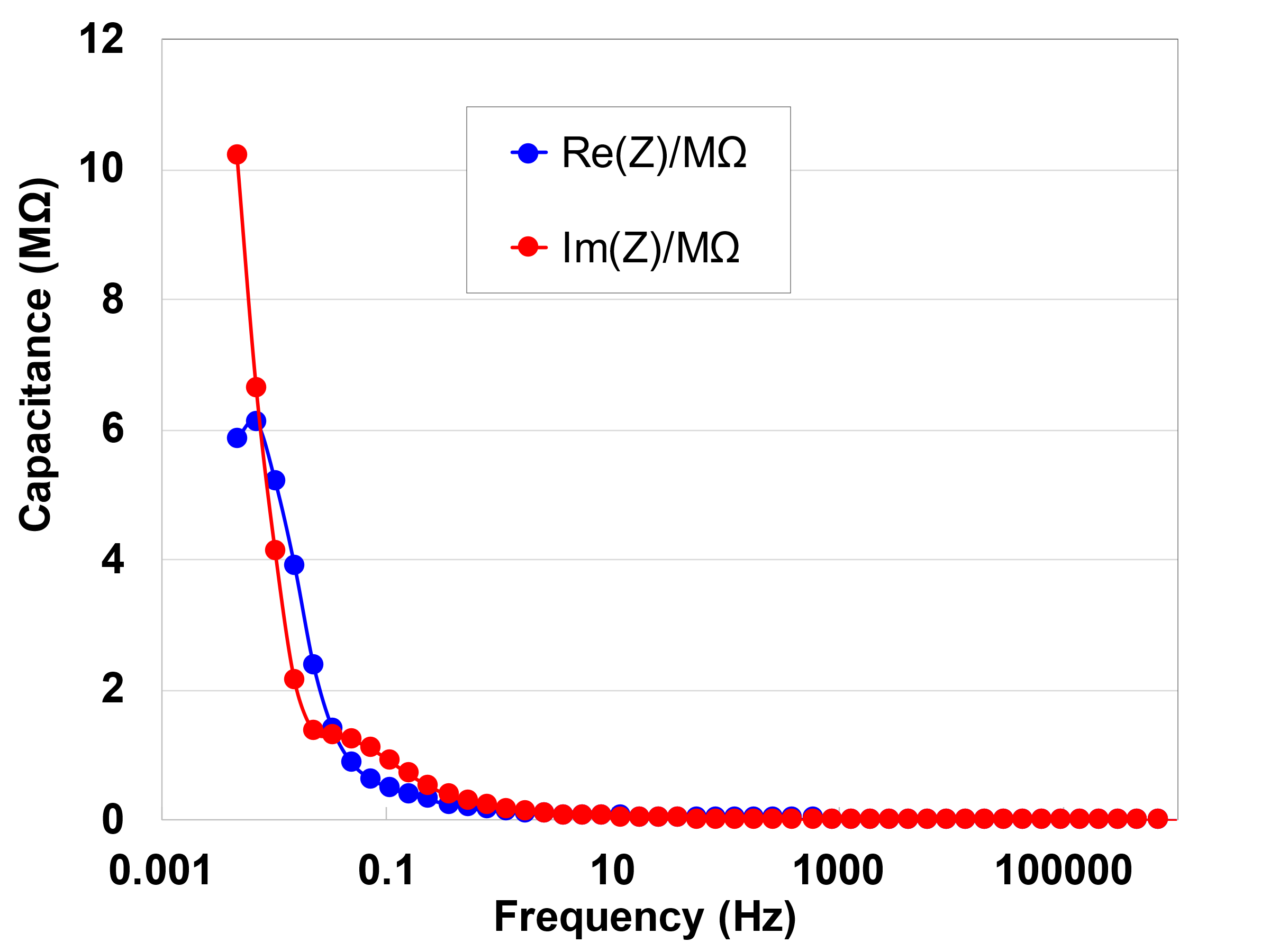


Fig. S5. Real and imaginary impedances as a function of frequency for the Na-ACF device.


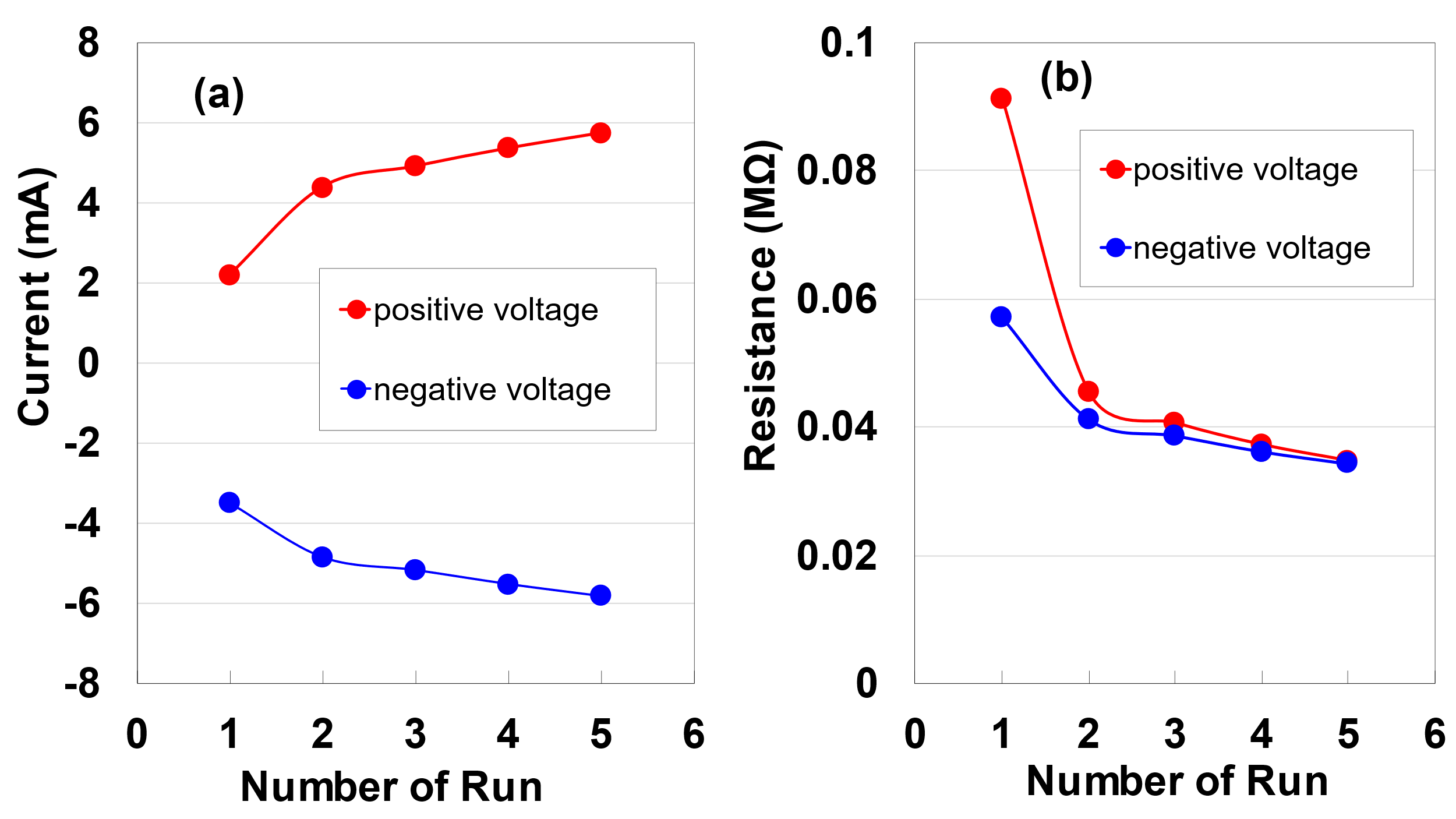


Fig. S6 The current and resistance at ± 200 V for *I ̶ V* and *R ̶ V* characteristics between -200 and + 200 V up to 5 times. (a) Current vs. number of times. (b) Resistance vs. number of times.

**S7. Comparison with AFC and the conventional cells**

Because the electric charges are absorbed on the the film surface of the Na-ACF, we must evaluate power density and energy density as W/m^2^ and Wh/m^2^, respectively. However, power density of W/m^3^ and energy density of Wh/m^3^ are practically used in conventional wet cells such as EDLCs and LIBs. In comparison with the conventional one, we calculated power density of W/m^3^ and energy density of Wh/m^3^ for the specimens stored at 2 mA and ~450 V, using its depth. Thus, we obtain power density of (700  *̶* 1950) W/kg and energy density of (0.1 *̶* 1.6) Wh/kg, when the depth and density are (0.4  *̶* 10) μm and 1.5 cm/g, respectively. The Ragone plot, the relation between energy density and power density, for the ACF supercapacitor is presented in Fig. 3d, along with conventional capacitors, EDLCs, and the second and fuel cells^14^. The plot of the ACF supercapacitor is located at capability area of EDLC. Further gains might be attained by use of thinner sheet and application of higher voltage. One enhancement way for electric storage ability is device fabrication by a nano-electromechanical system (NEMS) (see Fig. S10 in Ref. 5). Our results show that the Na- ACF specimen with lower density of 1.5 Mg/m^3^ is an ideal candidate for supercapacitors with potential applications of light electricity such as handheld electronic devices, transportation, and renewable energy storage for power grids^５^.

**S8. Rough estimate of specific surface area of the ACF supercapacitor**

Supercapacitor is characterized by use of porous electrode with specific surface area over 2,000 m^2^/g and formation of electric double layer (EDL). The porous electrode of conventional supercapacitors contacts to liquid electrolyte with a high probability, and the EDL is formed at boundary between the electrode and the electrolyte. In this case, the specific surface area of the porous electrode can be determined by BET method. On the other hand, electric charges in ACF supercapacitor used in this study are stored at convex nanometre-sized portions of the ACF, and formation of the EDL is attained by pairing of electron and proton at green area of convex surface in Fig. 4a. However, the ACF sheet is electrically contacted on the Al substrate by electrophoresis so that the ACF becomes close compact body. This means the ACF sheet seems to shrink in the density of the dense. Strictly speaking, the Al electrode do not contact to all convex points, *i.e*., arcs of fibres of CNF sheet due to connection between solid (Al) and solid (CNF), in comparison with connection of solid (porous carbon) and liquid electrolyte. The amounts of stored energies in the ACF supercapacitor depend on degree of contact between Al electrode and convex portion. However, we cannot measure directly the specific surface area of connected Al portions or convex portions of ACF sheet. Since the amount of electric charge roughly depends on the specific surface area, the stored energy serves conveniently as a measure of degree of contact between Al electrode and convex portion in place of specific surface area. Discharging behaviours for mechanical clump and electrophoresis methods is presented at Fig. S7. The stored energies of the former and the latter are 7.47 ×10^-8^ and 1.16 ×10^-6^ J/cm^2^, respectively. From 325 m^2^/g of CNF used in ACF device fabricated by mechanical method, we can roughly calculate CNF’s specific surface area of 5,000 m^2^/g (= 325 × 15.1). Thus, we assume that the ACF device connected on the Al electrode by electrophoresis method is supercapacitor.


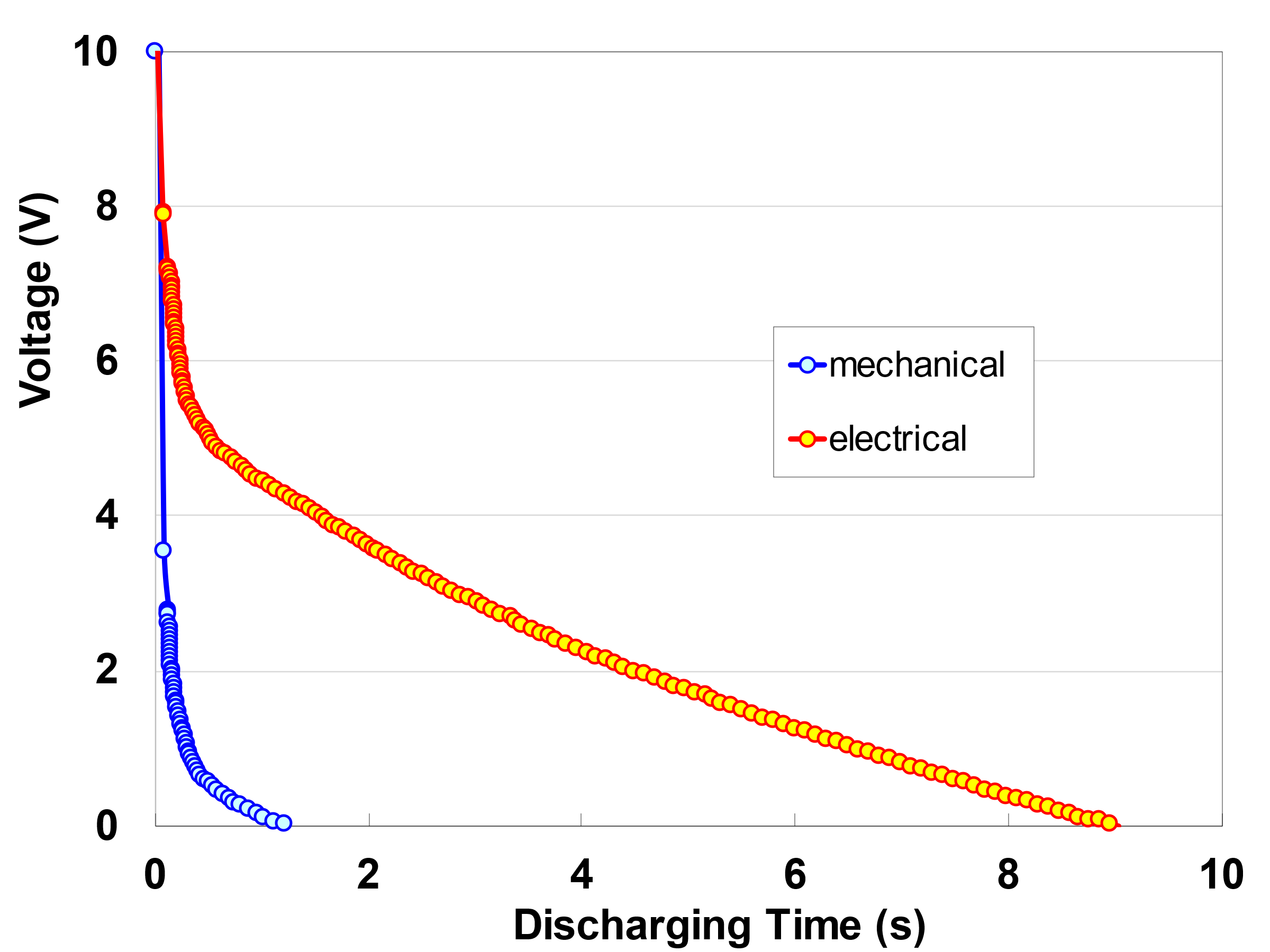


Fig. S7 Discharging behaviours for mechanical clump and electrophoresis methods after 1mA-10 V charging for 50 sec.

**S9. Electric storage inspection of the ACF supercapacitor**

To provide visible proof for electric storage of the ACF supercapacitor, we observed a lighting of white LED. The schematic experimental system is presented in Fig. S8, which is composed of regulated DC power supply and LED.

Fig. S8　Experimental inspection view for electric storage by LED lighting.

**S10.　Effect of voltage application**

As can be seen from Fig. 1b and d, the voltage application to the Na-ACF provides increase in electric charge and shortening in charging time as compared with conventional “wet” cells, such as EDLCs and LIBs which take a long time to charge under constant current^9^. Although power density of 0.479 W/m^2^ stored at 500 V (Fig. 1d) is around 22 times larger than that of 0.022 W/m^2^ stored at 10 V (Fig. 1b), energy density of 382.8 mJ/m^2^ stored at 500 V is around 2.8 times larger than that of 139.3 mJ/m^2^ stored at 10 V. This shows that voltage application to the Na-ACF has a large effect on power density and a small one on energy density. We must tackle a controlled problem of surface sheets with nanometre-sized uneven patterns for enhancement of energy density.

**S11.　Necessary conditions for superior electric energy storage**

We demonstrated that the ACF supercapacitor showed five necessary conditions for superior electric energy storage: 1. Quantum-size effect with nanometer-size uneven surface; 2. Electroadsorption effect by higher work function; 3. Offset effect of charge by positive radicals; 4. Integrating effect of charges by EDC circuit; and 5. Electric storage effect by electric double layer composed of solid (electrode)/solid (electrolyte) charge storage.

**References**

^22.^ Fukuhara, M., Fujima, N., Oji, H., Inoue, A. & Emura, S. Structures of the icosahedral clusters in Ni-Nb-Zr-H glassy alloys determined by first-principles molecular fynamics calculation and XAFS measurements, *J. Alloy Comp*., **497**, 182‒187 (2010). http://doi:10.1016/j.jallcom.2010.02.188

^23.^ Fujima, N., Hoshino, T. & Fukuhara, M. Local structures and structural phase change in Ni-Zr-Nb glassy alloys composed of Ni5Zr5Nb3 icosahedral clusters, *J. Appl. Phys*., **114**, 063501 (2013). http://dx.doi.org/10.1063/1.4817500

^24.^ Ishimaru, M. & Nakamura, R., Low temperature crystallization of amorphous materials by electron excitation effects, *J. Crystal. Soc. Jpn*, **61**, 29-34 (2019).

^25.^ Saito, T., Kimura, S., Nishiyama, Y. & Isogai, A. Cellulose nanofibers prepared by TEMPO-mediated oxidation of native cellulose, *Biomacromolecules* **8**, 2485‒2491 (2007).

^26.^ Yang, C. *et al*. Copper-coordinated cellulose ion conductors for solid-state batteries, *Nature*, **598**, 590‒596. <https://doi.org/10.1038/s41586-021-03885-6> (2021).
